# Biomimicking properties of cellulose nanofiber under ethanol/water mixture

**DOI:** 10.1038/s41598-020-78100-z

**Published:** 2020-12-03

**Authors:** Abdul Halim, Kuan-Hsuan Lin, Toshiharu Enomae

**Affiliations:** 1 Graduate School of Life and Environmental Sciences, University of Tsukuba, Tsukuba, Ibaraki 305-8572 Japan; 2 Faculty of Life and Environmental Sciences, University of Tsukuba, Tsukuba, Ibaraki 305-8572 Japan; 3 Department of Pulp and Paper Technology, Institute of Technology and Science Bandung, Jl. Ganesha Boulevard Lot-A1 Kota Deltamas, Cikarang Pusat, Bekasi, Bekasi, 17530 Indonesia; 4 Department of Chemical Engineering, Universitas Internasional Semen Indonesia, Jl. Veteran, Sidomoro, Kebomas, Gresik, 61122 Indonesia

**Keywords:** Bioinspired materials, Bioinspired materials

## Abstract

The two types of cellulose nanofiber (CNF) surface characteristics were evaluated by oil contact angle under ethanol–water solution at several concentrations as well as in air. Wood pulp-based 2,2,6,6-tetramethylpiperidine-1-oxylradical (TEMPO)-oxidized cellulose nanofiber (TOCNF) sheets and bamboo-derived mechanical counter collision cellulose nanofiber (ACC-CNF) sheets were fabricated by casting followed by drying. The CNF shows underwater superoleophobic mimicking fish skin properties and slippery surface mimicking Nepenthes pitcher. The underwater superoleophobic properties of CNF was evaluated theoretically and experimentally. The theoretical calculation and experimental results of contact angle showed a large deviation. The roughness, zeta potential, and water absorption at different concentrations were key factors that determine the deviation. Antifouling investigation revealed that CNF was a good candidate for antifouling material.

## Introduction

Fish skin have been already known for its properties to repel a liquid and solid fat from its surface. The properties known as a superoleophobic surface come from the hydrophilic protein of fish skin that absorbs much water. The water moieties on the surface will repel any non-polar liquid such as oil^1,2^. A cellulose nanofiber with abundant of hydroxyl groups on the surface is a candidate for underwater superoleophobicity. A numerical study on the oleophobic characteristics of hydroxyl functional groups clarified that hydroxyl groups showed strong underwater oleophobicity than other functional groups^3^. The hydroxyl group of cellulose will absorb much water and produce a water layer that repel oil.

The underwater superoleophobicity of a surface is generally measured in terms of the underwater oil contact angle. This contact angle is known to be predicted from modified Young’s equation, Wenzel equation or Cassie–Baxter equation, depending on the condition of the surface and attached oil droplets (see theoretical section in detail). However, the chemical properties and roughness of cellulose sheet surfaces are susceptible to an underwater environment. For instance, carboxylic groups of wood pulp-based 2,2,6,6-tetramethylpiperidine-1-oxylradical (TEMPO)-oxidized cellulose nanofiber (TOCNF) dissociate in underwater conditions. This tendency leads to an error of prediction. Even though the prediction of underwater contact angle have been reported^4–6^, there are few studies dealing with a numerical prediction characteristic of underwater oleophobicity of cellulose. Previous studies on the oleophobic property of cellulose mainly focused on filter applications^7–13^.

Herein, we report the contact angle prediction of cellulose nanofiber-coated surfaces under several concentrations of ethanol–water solutions and compare it to the experimental results. Two kinds of CNF: chemically-modified TEMPO-oxidized CNF (TOCNF) and mechanically modified CNF (ACC-CNF) are characterized and compared. Contrary to ACC-CNF containing only hydroxyl groups, TOCNF contains carboxylic groups on its surface. The effect of hydroxyl and carboxylic groups contained by CNF was clarified. The method of predicting the under-water contact angle using ethanol–water solutions is successfully established as an important information for designing an antifouling filters.

### Theory

In in-air condition, on a smooth surface, contact angles are predicted by Young’s equation^14^:

1 $$\mathrm{cos}{\theta }_{OA}=\frac{{\gamma }_{SA}-{\gamma }_{OS}}{{\gamma }_{OA}},$$

where $\gamma$ is the surface tension, $\theta$ is Young’s contact angle and subscripts $O$, $A$, and $S$ refer to oil, air and solid, respectively. The surface tension of oil is usually lower than that of water. According to Young’s equation, the contact angle of oil is lower than that of water; therefore, in air, a hydrophilic surface (water contact angle < 90°) is also oleophilic (oil contact angle also < 90°), and an oleophobic surface (oil contact angle > 90°) is also hydrophobic (water contact angle > 90°)^15^. For a rough surface, the interaction between the liquid and solid is emphasized. The contact angle of the liquid on a rough surface is described by Wenzel’s equation^16^:

2 $$\mathrm{cos}{\theta }_{OA}^{W}=r\mathrm{ cos}{\theta }_{OA},$$

where ${\theta }_{OA}^{W}$ is the Wenzel contact angle of a liquid on a rough surface. $r$ is the roughness defined as a ratio of the apparent surface area (${A}_{a}$) to the projected area (${A}_{s}$) as represented by equation:

3 $$r=\frac{{A}_{a}}{{A}_{s}}.$$

This condition is called the Wenzel state. If an inert fluid fills the void of a rough surface, the interaction between the liquid and solid is reduced. In this case, the contact angle is predicted by Cassie–Baxter’s equation^17^:

4 $$\mathrm{cos}{\theta }_{OA}^{CB}={f}_{so}\left({R}_{f}\mathrm{cos}{\theta }_{OA}+1\right)-1,$$

where ${\theta }_{OA}^{CB}$ is the Cassie–Baxter contact angle of a liquid on a rough surface. ${f}_{so}$ is the fraction of liquid–solid contact area to the total surface area and ${R}_{f}$ is the roughness of only the wetted area of the solid. In a water environment, Eq. (1) is complemented by relationships:

5 $${\gamma }_{SA}={\gamma }_{WS}+{\gamma }_{WA}\mathrm{cos}{\theta }_{WA}$$

6 $${\gamma }_{SW}={\gamma }_{OS}+{\gamma }_{OW}\mathrm{cos}{\theta }_{OW},$$

where subscript $W$ refers to water. Rearrangement of Eqs. (1), (5) and (6) leads to Eq. (7)^18^:

7 $$\mathrm{cos}{\theta }_{OW}=\frac{{\gamma }_{OA}\mathrm{cos}{\theta }_{OA}-{\gamma }_{WA}\mathrm{cos}{\theta }_{WA}}{{\gamma }_{OW}}$$

From Eq. (7), we could predict the underwater contact angle of the liquid if the in-air contact angle is given and Eqs. (2) and (3) are applied to calculate Wenzel’s and Cassie–Baxter’s contact angles, respectively, corresponding to the roughness condition.

## Results and discussion

### Surface structure of cellulose nanofiber

**Figure 1:**
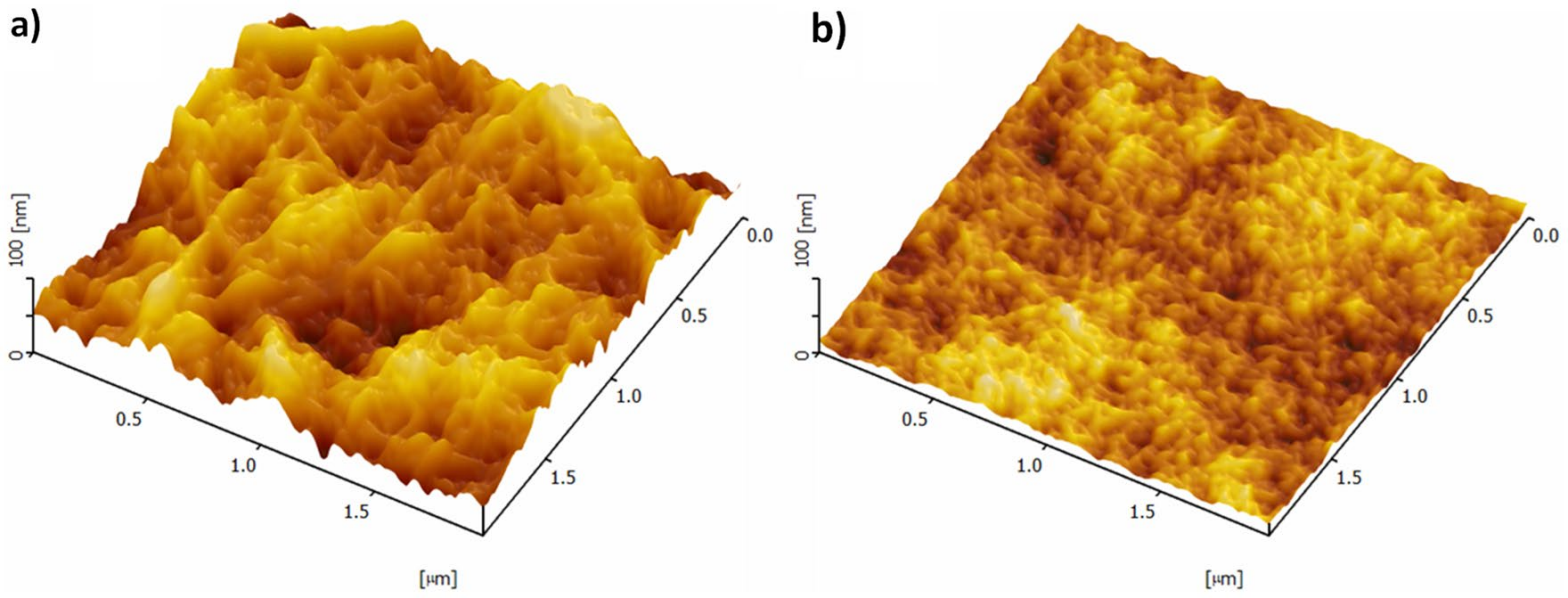
AFM images of ACC-CNF (**a**) and TOCNF (**b**) thin films.

Figure 1 shows that ACC-CNF and TOCNF have different textures and apparent surface roughness levels in the same area (2 µm × 2 µm) scanned by AFM. The apparent surface area (${A}_{a}$) for ACC-CNF and TOCNF are 4.253 µm^2^ and 4.005 µm^2^, respectively. The projected area (${A}_{s}$) for ACC-CNF and TOCNF both are 4 µm^2^. Therefore, the *r* values of ACC-CNF and TOCNF were 1.071 ± 0.01 and 1.002 ± 0.001, respectively. The difference of roughness is relatively small even though the feature of ACC-CNF and TOCNF is quite different as shown in Fig. 1 because the apparent surface area is relatively similar. The calculation and illustration of this phenomena are described in Figure S1. This difference in *r* is due to the fiber dimensions. Previous reports mentioned that ACC-CNF is 15–20 nm and *ca.*1 µm in diameter and length, respectively while TOCNF is 3–4 nm and < 3 µm^19,20^.

**Figure 2:**
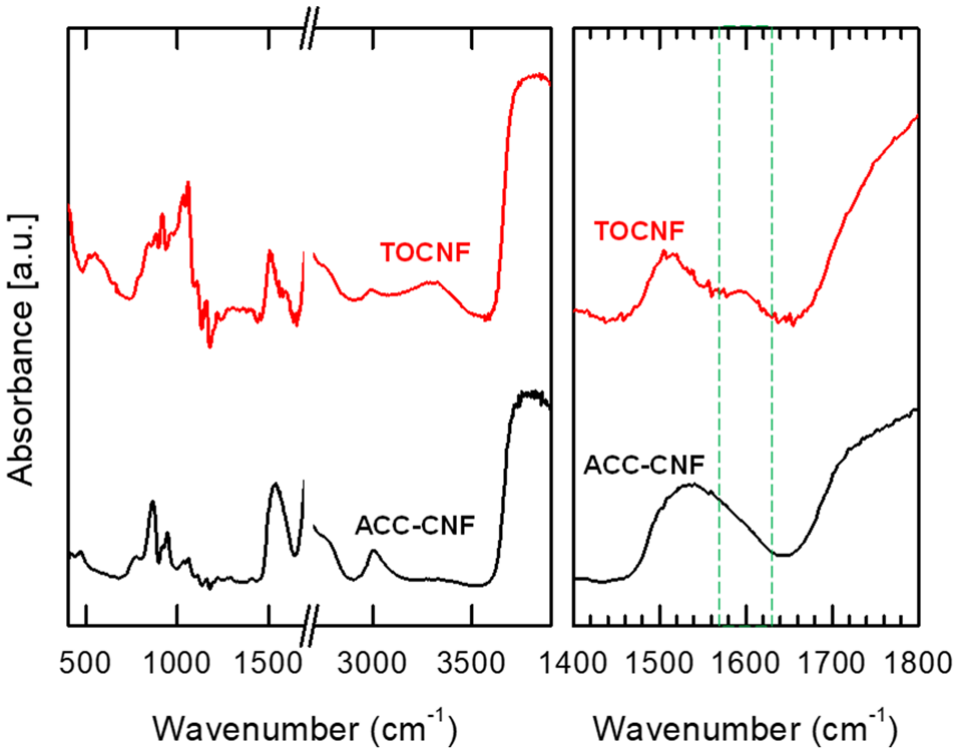
FTIR spectra of TOCNF and ACC-CNF.

FTIR spectra are depicted in Fig. 2. The absorption band at 3000–3600 cm^−1^ is due to hydroxyl groups of cellulose and water adsorbed on the cellulose. At the absorption band of 1610 cm^−1^, TOCNF shows an absorption peak assigned to the C=O stretching band of COONa. This bond is generated only on TOCNF during neutralization with sodium hydroxide^19^. The chemical reaction of TOCNF fabrication is described in the Figure S2.

### Oil and water contact angles on CNF surface under in-air condition

**Figure 3:**
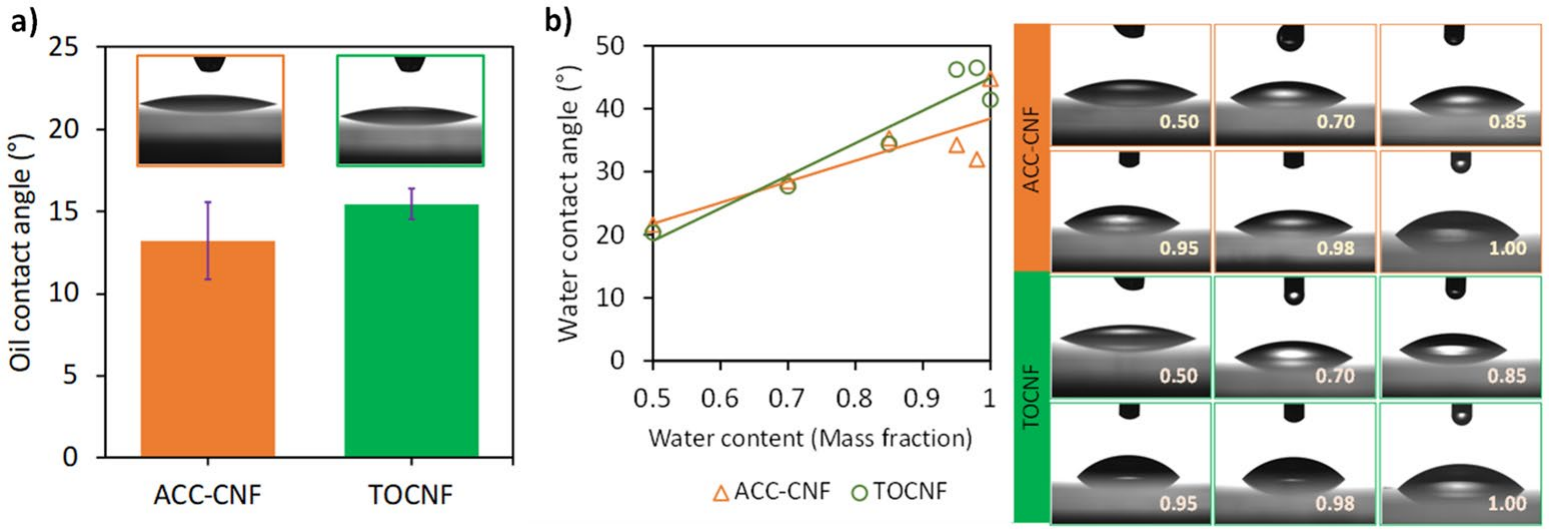
In-air contact angle of oil (**a**) and ethanol–water solutions (**b**) on CNF sheet surfaces with photographs. Values in the photographs in (**b**) refer to water content.

In air, TOCNF exhibited a higher oil contact angle (15.5°) than ACC-CNF (13.3°) as shown in Fig. 3. For TOCNF, hydroxyl groups in the cellulose chain were oxidized to carboxylic groups^19^. Carboxylic groups increase hydrophilicity because this functional group brings ionic bonds with sodium. A high element ratio of oxygen to carbon showed that hydrophilicity of the surface increased^21–23^. However, it is difficult to justify the contribution of the molecular structure of CNF to the contact angle since the contact angle is dependent on several other variables such as roughness. If a droplet in the Wenzel state is assumed, Young’s contact angle can be calculated from the roughness of TOCNF and ACC-CNF as the AFM result using Eq. (2). For ethanol–water solutions, a higher water content produced a higher contact angle because of its higher surface tension, as is consistent with Young’s equation. The surface energy of TOCNF and ACC-CNF was 56.96 and 51.81 mJ m^−2^, respectively, by the Fowkes equation and 43.67 and 44.14 mJ m^−2^, respectively, by the Owens–Wendt equation (refer to the Supplementary Information section B and Figure S3 in detail). This result agrees with the previous report referring to the cellulose surface energy of approximately 33–74 mJ m^−2^ ^24^.

### CNF properties underwater condition

**Figure 4:**
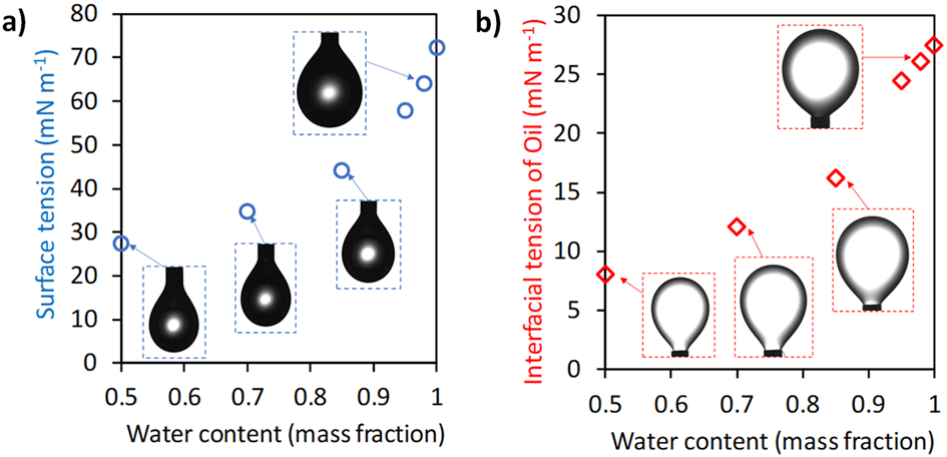
Surface tension of ethanol–water solution (**a**) and underwater interfacial tension of *n*-tetradecane as oil (**b**).

Water has a higher surface tension than oil and ethanol. Figure 4a shows the surface tension of water. In the pendant method, the accuracy of measurement was dependent on the pendant volume used during measurement. To obtain high accuracy, the pendant volume was increased to be large enough immediately before its detachment. A higher content of ethanol more largely decreased the surface tension because ethanol molecules break hydrogen bonds among water molecules. The result was in good agreement with the previous report^25^. A high content of ethanol also decreased the interfacial tension between oil and ethanol–water solution as shown in Fig. 4b. Ethanol with non-polar groups such as hydrocarbon chains presumably interacts with an oil droplet, causing a decrease in the surface energy.

**Figure 5:**
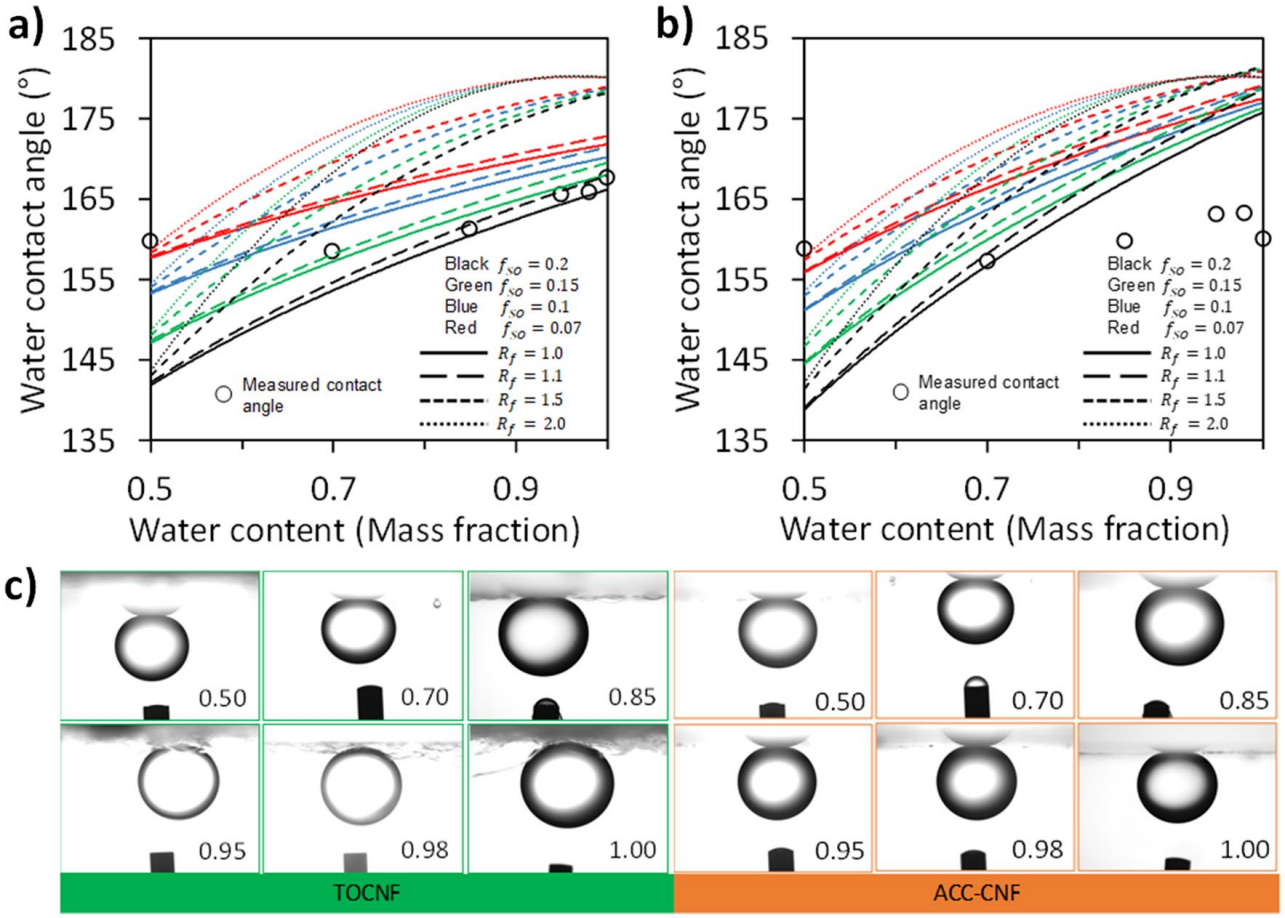
Theoretical and measured contact angles from images of oil under ethanol–water solutions on TOCNF (**a**) and ACC-CNF (**b**) surface with images of oil droplets at each water content (**c**).

By modifying Young’s equation in-air and underwater as shown in Eqs. (5–7), the underwater oil contact angle was calculated as shown in Fig. 5. The surface tension of *n*-tetradecane is 26.56 mN m^−1^^26^. The in-air oil contact angle, in-air ethanol–water solution contact angle, and interfacial surface tension between oil and ethanol–water solution were obtained from images in Fig. 3. The theoretical calculation of underwater contact angle is shown in Fig. 5a,b for TOCNF and ACC-CNF, respectively and its photograph image (Fig. 5c). Higher ${R}_{f}$ will increase significantly the contact angle especially at high water contents (further geometry is explained in Supplementary Information section C, Figure S4). At 50% water, the contact angle is similar for several ${R}_{f}$ value.

In air, from Young’s equation and as shown in Fig. 3, the hydrophilic surface is also oleophilic due to the surface tension of oil usually lower than water. On the Cassie–Baxter state, the hydrophilic surface is possible to show high apparent contact angles (> 90°) because that are air fill the void on the rough surface. The air will decrease the surface interaction of solid and liquid on the interface. A high ${R}_{f}$ value means high solid–liquid interaction. Therefore, under air condition, apparent contact angle will decrease. Underwater condition, the hydrophilic surface change to oleophobic because water will fill the void on the rough surface. However, in Fig. 5a,b, a higher ${R}_{f}$ value does not show a lower oil contact angle even though the solid-oil interaction is stronger. The phenomena might be caused by the oleophobicity of the CNF surface that increases significantly with increasing water content and not because of the roughness of surface.

Underwater condition, ${f}_{so}$ represents the fraction of void filled with water or water–oil interaction on the rough surface. A high ${f}_{so}$ value means a low void fraction or strong solid-oil interaction on the rough surface so that high ${f}_{so}$ values will produce low oil contact angles as shown in Fig. 4a,b. However, experimental underwater oil contact angles were always underwater superoleophobic (> 150°) as shown in Fig. 5a,b. The large difference between theoretical calculation and experimental results are derived from the theoretical equation that does not consider the change of underwater surface properties chemically or physically.

**Figure 6:**
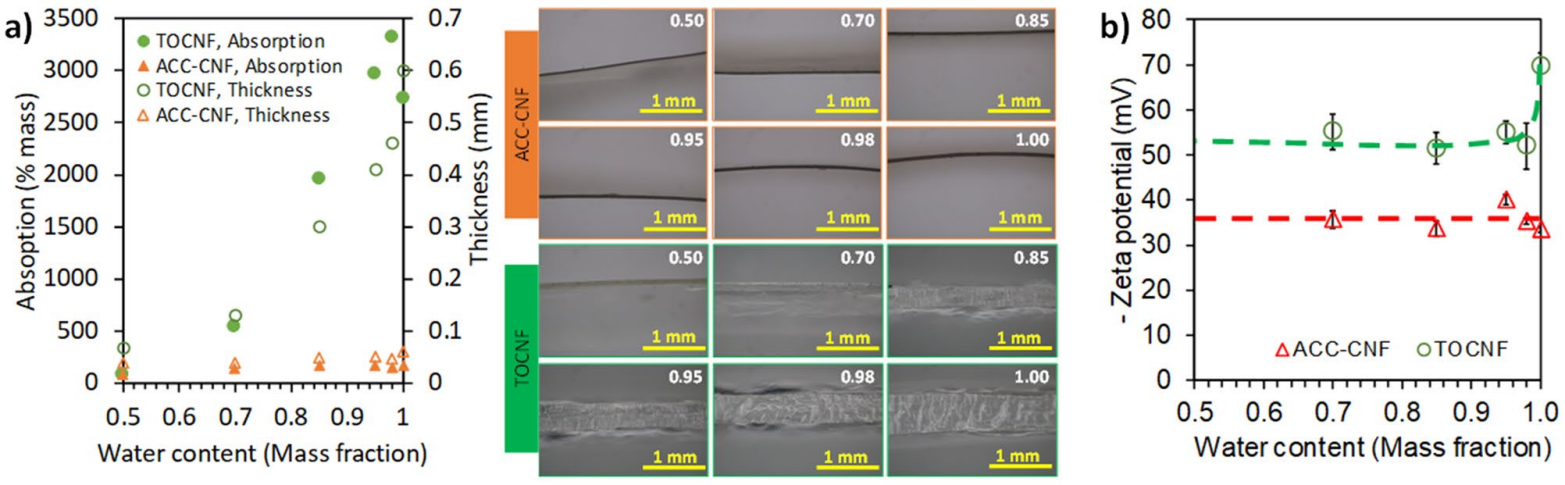
Absorption capacity (**a**) and zeta potential (**b**) of cellulose nanofiber.

As shown in Fig. 6a, cellulose sheets absorbed so much water that the surface roughness and hydrophilicity increased. TOCNF absorbed more water than ACC-CNF presumably due to its higher hydrophilicity. The thickness and absorption capacity increased exponentially for TOCNF and linearly for ACC-CNF as a function of water content. The physical appearance of TOCNF after absorbing water was gel-like, whereas that of ACC-CNF after absorbing water was rubber-like. The swelling capacity of TOCNF and ACC-CNF was 2050% and 256%, respectively^10^. However, the absorption capacity did not agree with the contact angle qualitatively, as shown in Fig. 6a. It is presumably because at higher water contents, the surface had higher roughness than that at lower water contents.

Although the contact angle is macroscopic behavior, the report indicated that the contact angle is affected by nanoscale molecularly roughness of surface^27^. Therefore, another factor possibly affecting the deviation is zeta potential of CNF. Zeta potential indicates the surface charge of CNF that affects the contact angle^28,29^. TOCNF had a zeta potential twice as high as ACC-CNF as shown in Fig. 6b because TOCNF contains carboxyl groups. In underwater condition, CNF surfaces formed an electric double layer and induced a high zeta potential. For TOCNF, the zeta potential decreased after ethanol addition in agreement with the previous report^30^ as more carboxylic groups associated at lower water contents. However, no difference in the dependence of the zeta potential on the water content was found for ACC-CNF. This result indicates that the surface charge of ACC-CNF in ethanol–water solutions was not affected by the water content as ACC-CNF only has hydroxyl groups. Considering that an oil droplet has slightly negative charge in water^31^, the negative zeta potential leads the electrostatic repulsion between a CNF surface and oil; however, this concept is not considered in Eq. (7). Higher levels of zeta potential and absorption capacity of TOCNF are supposed to cause higher underwater oil contact angle. Underwater, TOCNF showed a property like fish mucus. On fish scales, mucus keeps the fish skin always clean even under dirty water^32^.

### Application of superoleophobic properties of cellulose nanofiber sheet

**Figure 7:**
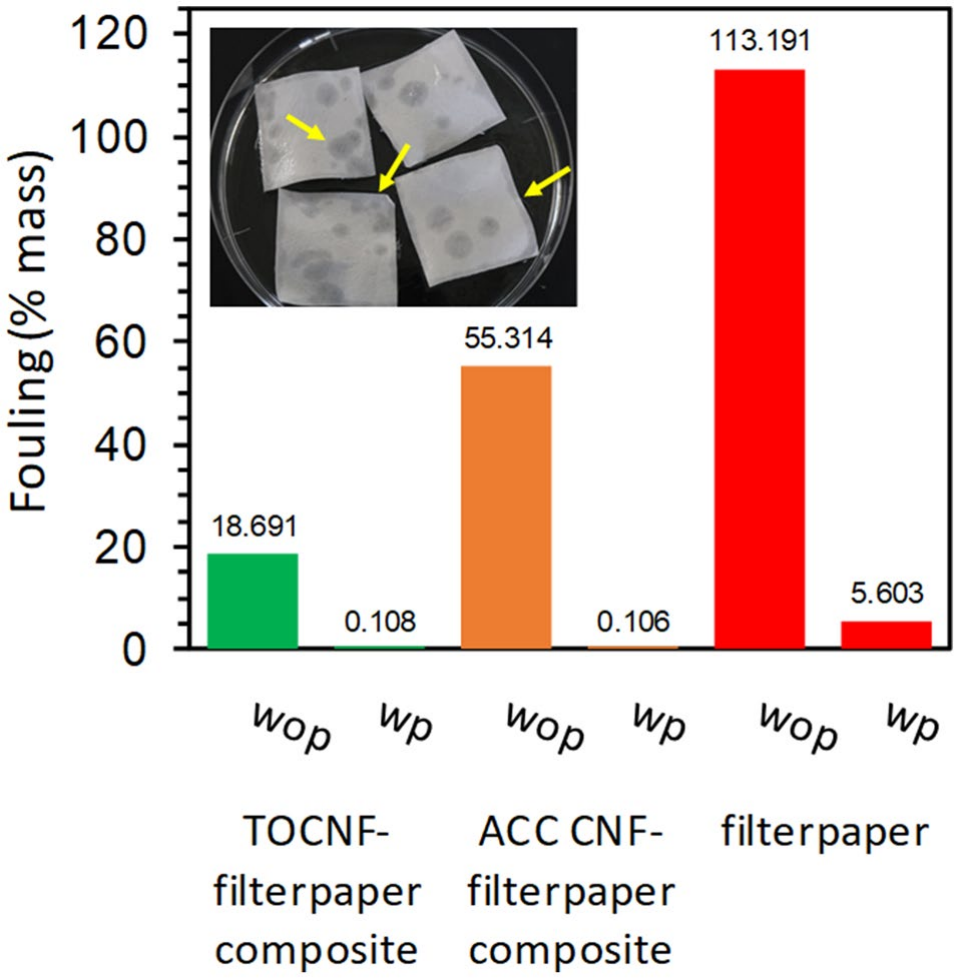
Fouling level of CNF-FP sheets with (wp) and without (wop) prewetting with water.

The ability to keep water that mimic a mucus fish scale underwater leads CNF sheets with underwater oleophobic properties seem promising for antifouling applications^7,8,33,34^. For prewetting, only a small amount of oil was absorbed as shown in the picture of Fig. 7. The oil droplet slid on the surface due to the repellency developing in the porous structure of nanocellulose sheets containing a lot of water molecules. Absorbed oil might cause damage to the surface, edge or boundary of samples. Without prewetting, the sample absorbed oil yet less than filter paper. This is because CNF forms a tighter structure and decreases the porosity. Interestingly, the oil rejection works not only underwater but also in air. The in-air oil rejection property is similar to that of a Nepenthes pitcher surface^35^. This indicates that CNF is mimicking not only fish skin but also Nepenthes pitcher. Compared to the Nepenthes pitcher mimicking surface reported by Guo et al.^36^ and Chen et al.^37^, the CNF surface can be applied as an oil repellant material without any further chemical modification.

## Materials and methods

Two types of cellulose nanofiber, wood pulp-based 2,2,6,6-tetramethylpiperidine-1-oxylradical (TEMPO)-oxidized cellulose nanofiber with a carboxylate content of 0.016 mmol/g^19^ (TOCNF, Nippon Paper Industries Co., Ltd., Japan) and bamboo derived mechanical counter collision cellulose nanofiber^20^ (ACC-CNF, Chuetsu Pulp & Paper Co., Ltd., Japan), was used. One mL of CNF coated on a glass slide using spin coater (Mikasa Opticoat MS-B100) with 60 rpm for 1 min. The glass slide (Matsunami S1111, Japan) was cleaned by immersion in sulfuric acid for at least for 2 days in advance. The CNF coated glass then dried at 65 °C for 24 h in an oven (Sanyo MIR-262, Japan). CNF sheets were fabricated by casting 5, 7, and 10 g of a CNF suspension in a petri dish with a diameter of 65.83 mm then drying it at 65 °C for 24 h. 1% weight TOCNF aqueous suspension was stirred for at least 24 h until the TOCNF suspension changes from gel-like to a liquid form. 1.35% ACC-CNF aqueous suspension was diluted to 1% then stirred for at least 24 h.

CNF-coated filter paper (CNF-FP) was fabricated by immersion of filter paper (Advantec No. 185) in the CNF dispersion for 30 min followed by drying at 65 °C for 24 h. To measure the water content, CNF-FP was dried at 120 °C for 48 h. Mass of CNF-FP was measured before and after coating. The thickness of the sheets was measured with a micrometer (TW-21 Tozai Seiki Co., Ltd., Japan). Fourier Transform Infrared (FTIR) spectra of the TOCNF and ACC-CNF sheets were measured to analyze functional groups with a spectrometer (Shimadzu IR Prestige 21, Japan).

Oil contact angle (OCA) and water contact angle (WCA) measurements were carried out at CNF coated glass surface by the sessile drop method (DropMaster DMs-401, Kyowa Interface Science Co., LTD., Japan). The contact angle was measured in air and under ethanol–water solution. n-tetradecane (Wako Pure Chemical Industries Ltd., Japan) and deionized water were used for OCA and WCA measurements, respectively. The theoretical calculation method of OCA is described in Supplementary Information material section A. The weight ratio of water to ethanol (Special grade, Wako Pure Chemical Industries Ltd., Japan) was 0.50, 0.70, 0.85, 0.95, 0.98 and 1.00. The surface tension of the ethanol water solution and the interfacial surface tension between oil and the ethanol–water solution were measured by the pendant drop method (DropMaster DMs-401, Kyowa Interface Science Co., LTD., Japan).

Swelling capacity of the CNF sheets was measured by soaking a CNF sheet in ethanol–water solution for at least 10 min until saturation and expressed in the contained solution mass on dry cellulose mass in percentage. The thickness of swollen CNF sheets was measured from pictures taken with a microscope (SZX10, Olympus, Japan) using open source software ImageJ^38^. Atomic Force Microscopy (AFM, Hitachi High-Tech Science E-Sweep, Japan) was applied to observe the topography and roughness of CNF sheets. The cantilever has a spring constant of 125 N/m and vibrated at a frequency ranging 110–150 kHz, 225 µm in height with a probe of ≤ 10 nm in tip diameter and 256 resolution was used. The roughness *r* of the CNF sheets was calculated by Eq. (3). The apparent surface area (${A}_{a}$) was obtained from AFM and projected area is equal to scanned area of AFM. The effect of scanned area and resolution to the roughness was negligible as previously described by Kozbial et al.^39^. Zeta potential of CNF dispersion was measured by Electrophoresis and Dynamic Light Scattering (DLS) (Zetasizer Nano ZS, Malvern Instruments, UK).

Fouling analysis was conducted by dropping 5 ml of tetradecane on a CNF-filter paper (CNF-FP) sheet and it was allowed to stand for 30 min with or without a prewetting process that was carried out by immersion of a CNF-FP sheet in deionized water for 5 min. The fouling result was evaluated by drying the CNF-FP after fouling in oven at 75 °C for 12 h. The samples were weighed before and after drying.

## Conclusion

The cellulose nanofiber shows superoleophobic under various concentrations of ethanol water solution. However, the experimentally-measured and theoretically-calculated contact angles showed a high deviation presumably due to roughness and chemical properties of CNF surfaces that is subject to change in the aqueous solution with the length of wetting time. Changes of these properties are not considered in the theoretical calculation. In the solution, high water-swelling TOCNF sheets absorbed more water and showed a higher zeta potential than ACC-CNF. The amount of absorbed water, that is, accommodation capacity increased exponentially with the water content of the solution. Both CNF sheets exhibited in-air oleophobicity like a Nepenthes pitcher surface and TOCNF exhibited a mucus like underwater property. These antifouling results imply that CNF is capable of repelling oil and promising as an antifouling material.

## Supplementary information

Supplementary Information.
