# Supplementary material for: Biomimicking properties of cellulose nanofiber under ethanol/water mixture
Source: Sci Rep. 2020 Dec 3;10:21070. doi: 10.1038/s41598-020-78100-z (PMC7712784; doi:10.1038/s41598-020-78100-z)
Supplement: Supplementary file 1 — Supplementary Information. [file 41598_2020_78100_MOESM1_ESM.docx]

**Supplementary Information**

**Biomimicking Properties of Cellulose Nanofiber Under Ethanol/Water Mixture**

Abdul Halim^a,c,d*^, Kuan-Hsuan Lin^a^, Toshiharu Enomae^b*^

^a^Graduate School of Life and Environmental Sciences, University of Tsukuba, Tsukuba, Ibaraki 305-8572, Japan

^b^Faculty of Life and Environmental Sciences, University of Tsukuba, Tsukuba, Ibaraki 305-8572, Japan

^c^Department of Pulp and Paper Technology, Institute of Technology and Science Bandung, Jl. Ganesha Boulevard Lot-A1 Kota Deltamas, Cikarang Pusat, Bekasi, Jawa Barat, 17530, Indonesia

^d^Department of Chemical Engineering, Universitas Internasional Semen Indonesia, Jl. Veteran, Sidomoro, Kebomas, Gresik, 61122, Indonesia

*corresponding author:

abdul-halim.xm@alumni.tsukuba.ac.jp

enomae.toshiharu.fw@u.tsukuba.ac.jp

**Figure S1**. The illustration of surface area of ACC-CNF (a) and TOCNF (b)

Figure S1 shows difference feature however the result of roughness calculation is similar. The projected area of a) and b) is 5. The area of a) is 11 and of b) is 10.5. Therefore, the roughness is 2.2 and 2.1

1. **Contact angle calculation steps**
2. Under air water contact angle and under air oil contact angle were measured experimentally.
3. Young’s contact angles of the solid surface under air were calculated by dividing apparent contact angle to the rough surface of solid measured from AFM. The state was assumed to be Wenzel’s state.
4. Equation then applied to calculate theoretical underwater young’s contact angle.
5. Cos more than -1 or 1 is assumed or equivalented to -1 or 1 respectively.
6. Underwater Cassie-Baxter state contact angle then calculated with several wetted solid fraction.

**Figure S2**. Reaction mechanism of TOCNF fabrication [1]

1. **Surface Energy Prediction**

The surface energy of cellulose nanofiber sheets was predicted from Fowkes model [2] and Owens-Wendt model [3]. The surface energy ($\gamma_{SA}$) is divided into polar ($\gamma_{SA}^{p}$) and dispersive ($\gamma_{SA}^{d}$) components.

$\gamma_{SA}=\gamma_{SA}^{p}+\gamma_{SA}^{d}$ (S1)

1. The Fowkes model

The Fowkes equation is described as

$\frac{\gamma_{LA}\left( \cos\theta+1 \right)}{2}=\left( \gamma_{LA}^{d} \right)^{1/2}\left( \gamma_{SA}^{d} \right)^{1/2}+\left( \gamma_{LA}^{p} \right)^{1/2}\left( \gamma_{SA}^{p} \right)^{1/2}$ (S2)

where $\gamma_{LA}$ is liquid surface tension and $\theta$ is Young’s contact angle. First step, tetradecane as nonpolar liquid with $\gamma_{LA}^{p}=0$ was tested to determine $\gamma_{SA}^{d}$ from equation S2. Second step, water as polar liquid with $\gamma_{LA}^{d}=21.8$ mN m^-1^ and $\gamma_{LA}^{p}=50.95$ mN m^-1^ [4] was tested to determine $\gamma_{SA}^{p}$ from equation S3. The surface energy then calculated from equation S1.

1. The Owens-Wendt model

The Owens-Wendt equation is described as

$\gamma_{SL}=\gamma_{SA}+\gamma_{LA}-2\left( \gamma_{SA}^{d}\gamma_{LA}^{d} \right)^{\frac{1}{2}}-2\left( \gamma_{SA}^{p}\gamma_{LA}^{p} \right)^{\frac{1}{2}}$ (S3)

by combining Young’s equation, equation S3 is modified to

$\frac{\gamma_{LA}\left( \cos\theta+1 \right)}{2\left( \gamma_{LA}^{d} \right)^{1/2}}=\left( \gamma_{SA}^{p} \right)^{1/2}\frac{\left( \gamma_{LA}^{p} \right)^{1/2}}{\left( \gamma_{SA}^{d} \right)^{1/2}}+\left( \gamma_{SA}^{d} \right)^{1/2}$ (S4)

Plotting the left side of equation S4 against $\frac{\left( \gamma_{LA}^{p} \right)^{1/2}}{\left( \gamma_{SA}^{d} \right)^{1/2}}$ will produce linier data points. The dispersive and polar component of tetradecane, water and alcohol mixture were obtained from Ref [4]. Figure S1 shows linier plot of equation S4 for several liquids.

**Figure S3**. Owens-Wendt plots for ACC-CNF (a) and TOCNF (b) sheet.

1. **Derivation of Cassie-Baxter state equation**

**Figure S4**. geometry illustration of underwater Cassie-Baxter state

The apparent contact angle on rough surface with immiscible fluid fill the void of rough surface is predicted by [5]

$$\cos\theta_{CB}=f_{1}\cos\theta-f_{2}$$

where $f_{1}$ and $f_{2}$ is fraction of oil-wetted surface area and oil-air interface area, respectively. By applying $R_{f}$ as a ratio between oil-wetted surface area to the projected oil-wetted surface area ($R_{f}=\frac{f_{1}}{f_{SO}}$) and $f_{2}+f_{so}=1$, equation S can be written as

$$\cos\theta_{CB}=f_{so}R_{f}\cos\theta-\left( 1-f_{so} \right)$$

$$\cos\theta_{CB}=f_{so}\left( R_{f}\cos\theta+1 \right)-1$$

Ref.

1. Isogai, A., Saito, T. & Fukuzumi, H. TEMPO-oxidized cellulose nanofibers. *Nanoscale* **3**, 71-85 (2011)
2. Fowkes, F. M. Additivity of intermolecular forces at interfaces. I. determination of the contribution to surface and interfacial tensions of dispersion forces in various liquids. *J. Phys. Chem.* **67**, 2538-2541 (1963)
3. Owens, D. K. & Wendt, R. C. Estimation of the surface free energy of polymers. *J. Appl. Polym. Sci.* **13**, 1741-1747 (1969).
4. Janczuk, B., et al. The surface tension components of aqueous alcohol solutions. *Colloids and Surfaces* **36**, 391-403 (1989).
5. Cassie, A. B. D. & Baxter, S. Large contact angles of plant and animal surfaces. *Nature* **155**, 21-22 (1945).
